# GDP Forecasting: Machine Learning, Linear or Autoregression?

**DOI:** 10.3389/frai.2021.757864

**Published:** 2021-10-15

**Authors:** Giovanni Maccarrone, Giacomo Morelli, Sara Spadaccini

**Affiliations:** ^1^ Department of Economic and Social Sciences, Sapienza University of Rome, Rome, Italy; ^2^ Department of Statistical Sciences, Sapienza University of Rome, Rome, Italy; ^3^ Department of Methods and Models for Economics, Territory and Finance, Sapienza University of Rome, Rome, Italy; ^4^ Global Data Hub, Enel Global Services S.r.l., Rome, Italy

**Keywords:** k nearest neighborhood, machine learning, time series, GDP, forecasting strategies

## Abstract

This paper compares the predictive power of different models to forecast the real U.S. GDP. Using quarterly data from 1976 to 2020, we find that the machine learning K-Nearest Neighbour (KNN) model captures the self-predictive ability of the U.S. GDP and performs better than traditional time series analysis. We explore the inclusion of predictors such as the yield curve, its latent factors, and a set of macroeconomic variables in order to increase the level of forecasting accuracy. The predictions result to be improved only when considering long forecast horizons. The use of machine learning algorithm provides additional guidance for data-driven decision making.

## 1 Introduction

The real Gross Domestic Product (GDP) is a single, omni-comprehensive measure of the economic activity that considers the total value of goods and services produced in the economy. It is considered by academics, investors, and regulators as a proxy for the wealth of the economy and an informative indicator that drives the decision-making processes (Provost and Fawcett, 2013). This makes the forecast of the GDP a relevant issue. Indeed, it is of interest to target national economic policies as well as in other fields, from non-performing loans (Bouheni et al., 2021) to natural disaster (Atsalakis et al., 2020).

When the research question is the forecast of periods of growth or recession a popular methodology is to decompose the GDP in cyclical and trend components relying on appropriate filters. A growth (recession) means that the value of the cycle component is positive (negative) for a given period. However, within this approach it is only possible to assess the growth (or recession) losing the quantitative information on the prediction. This is instead achieved through the regression methodology. That it is the approach we follow, such choice being driven by the limitations encountered in the decomposition of the GDP in cyclical and trend components (Luginbuhl and Koopman, 2004).

The aim of this paper is to show among a set of different models and forecasting strategies which performs better. Classical time series analysis or machine learning? One-step-ahead or multi-step-ahead forecast? Including macro-economic variables or just the self-explanatory GDP values? How does our model respond to periods of economic turbulence? These are the research questions we aim to provide an answer.

Several approaches have been proposed in the literature to forecast the GDP. Indeed, the macroeconomic literature that investigates this topic through the time series approach mainly use different specifications of VAR (Ang et al., 2006; Brave et al., 2019; Koop et al., 2020), and forecasting improvements can be achieved relying on appropriate Bayesian shrinkage procedures, as highlighted in Bańbura et al. (2010). Regarding the potential economic indicators that are used as predictors of the GDP, many authors converge on the use of the yield curve that contains information about future economic activity (Giannone et al., 2008; Yiu and Chow, 2010). Estrella and Hardouvelis (1991) find that especially the slope of the yield curve can predict cumulative variations in real GDP for up to 4 years into the future. A similar study is carried out by Bernard and Gerlach (1998) in eight countries finding that, although there are substantial differences across the countries, the slope of the yield provides information about the possibility of future recessions, whereas Ang et al. (2006) find that nominal short rates outperform the slope of the yield curve in forecasting GDP growth. Other studies (Koop, 2013; Schorfheide and Song, 2015) use instead a set of macroeconomic variables to predict the U.S. GDP. Drawing from this strand of literature, e.g., Estrella and Mishkin (1996), Koop (2013), Diebold et al. (2006), we use the yield curve as well as its latent factors and a set of macroeconomic variables, namely Consumer Price Index, Unemployment rate, Federal Fund rates, and Manufacturing Capacity Utilization.

Chauvet and Potter (2013) offer a comparison between reduced form, autoregressive, VAR, and Markow switching models and find that simple time series autoregressive process of order two [AR (2)] outperforms other models in the forecast of the U.S. GDP. Baffigi et al. (2002) provide an example of the use of ARIMA for the U.E. GDP prediction. Lunde and Torkar (2020) exploit more than 120 predictors and then perform a principal component analysis (PCA) to reduce the number of variables. Despite the inclusion of different sources of information in their set-up, the PCA does not provide the economic interpretation of the results.

In this paper, we propose models with macro-economic variables and other models that take advantage of the self-explanatory information of the GDP relying on both classical time series analysis as well as on a machine learning algorithm. In particular, we forecast the U.S. GDP with ARX, SARIMAX and Linear Regression to include additional information such as real and financial measures of economic activity, and use AR and SARIMA as a benchmark for time series analysis. We also exploit the K-Nearest Neighbour (KNN) machine learning methodology. Our goal is to achieve forecasts with high accuracy and with high degree of explainability that is a best practice for building trust between machine learning and decision-makers, as pointed out in Bellotti et al. (2021). The idea is that the decision-maker should adopt the machine learning as a powerful instrument and should employ it with awareness without regarding it as a “black-box.”

Many studies explore the potential of machine learning in the field of forecasting. Stone (1977) shows the consistency property of the non-parametric KNN estimator. The model is widely used for classification tasks such as object identification and, due to the easy implementation and explainability, it is also used in applications such as missing data imputation (Bertsimas et al., 2021) and reduction of training set (Wauters and Vanhoucke, 2017) being able to better identify similar objects. The KNN can identify repeated patterns within the time series and for this reason is applied to financial time series modeling as in Ban et al. (2013). Al-Qahtani and Crone (2013) use KNN for forecasting U.K. electricity demand and find that KNN outperforms better forecasts than other benchmark models. Rodríguez-Vargas (2020) finds that KNN outperforms also two competitors machine learning models, the random forest and the extreme gradient boosting, in terms of accuracy for predicting the inflation. In general, KNN has been referenced as one of the top ten algorithms in data mining (Wu et al., 2008). Moreover, KNN is especially suitable for cases in which there is not an high number of past observations, i.e., very little past information. As pointed out in Wauters and Vanhoucke (2017), artificial intelligence methods require a minimum number of observation to work properly whereas for the KNN this limitation is not so strict even though a minimum number of observation is required (Diebold and Nason, 1990). We therefore employ the KNN model as it offers a simple methodology based on distance metrics to exploit past information.

A compelling way to predict real economic variables is offered by the nowcasting literature, which aims to predict their values in the very short term. When the objective is to study the prediction at horizons lower than a quarter, given quarterly data available for GDP, it is possible to use a consistent two step estimator, as in Doz et al. (2011), that provides the policymaker with an early estimate of the next quarter including auxiliary exogenous predictors available at a lower frequency. Moreover, this framework can be empowered with alternative variables to boost the economic knowledge. For instance, Spelta and Pagnottoni (2021) use nowcasting to assess the impact of mobility restrictions on the economic activity during the pandemic. In particular, they study the trade-off between economic sacrifices and health outcomes in terms of timely policy suggestions. Foroni et al. (2020) explicitly focus on the forecast and nowcast of COVID-19 recession and recovery studying the GDP growth and showing an interesting similarity with the great recession.

We analyze two different forecasting strategies: the one-step-ahead and the multi-step-ahead forecasts (Marcellino et al., 2006; Hu et al., 2020). The former is more reliable and accurate by construction, however it results to be less informative for macroprudential policies. In the multi-step-ahead strategy proposed, we forecast the U.S. GDP up to 12 quarters in advance. This information is potentially extremely valuable although much more challenging.

Finally, we evaluate the performance in terms of mean square error. In particular, we are interested in studying the trade-off between two different aspects: the accuracy of the estimates even when considering a period of economic turbulence, and the forecasting horizon.

The rest of the paper is organized as follows: Section 2 introduces the model specifications and the empirical strategy, Section 3 illustrates the empirical analysis, Section 4 reports the results and Section 5 concludes.

## 2 Model Specifications and Empirical Strategy

### 2.1 Motivations

**FIGURE 1:**
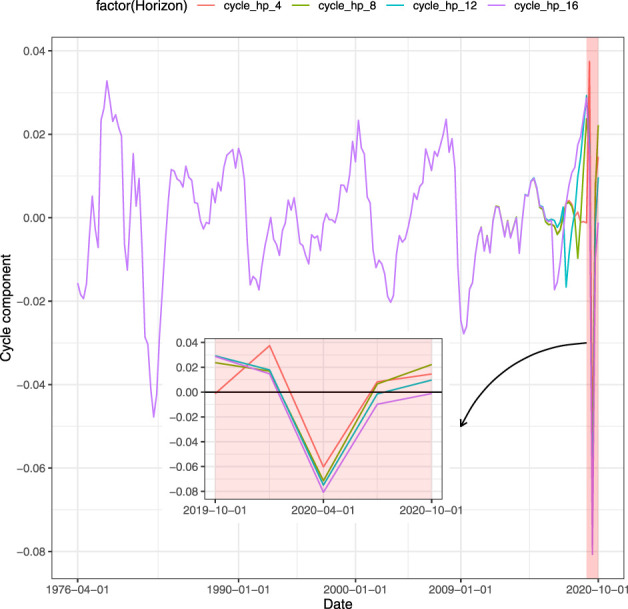
Cycle decomposition of GDP, sensitiveness to different horizons until last quarter of 2020.

**FIGURE 2:**
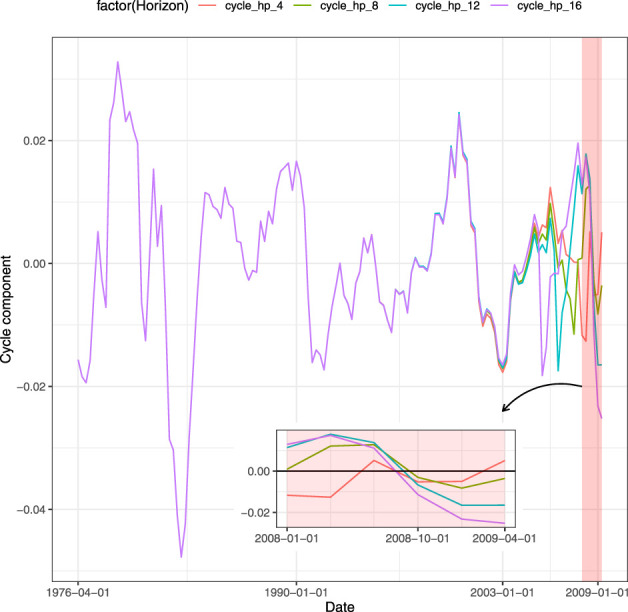
Cycle decomposition of GDP, sensitiveness to different horizons until second quarter of 2009.

Closely related to the GDP forecast is the ability to understand whether the forecasted value is associated with growth or recession for the economy. It can be achieved through a classification framework that defines a binary target variable starting from the time series of the GDP. An appealing approach to detect recessions is to decompose the GDP in trend and cyclical components. Among the techniques used, the filters are the most employed in literature. A well-known technique is the Hodrick and Prescott (1997) filter, also known as H-P filter, which through an appropriate parametrized minimization problem generates the GDP cycle component. Once the cycle component has been detected from the time series, it is then transformed into a binary variable that assumes value equal to 1 (recession) whenever the cyclical component is lower than zero and 0 (growth) otherwise. Nevertheless, the use of this approach has been criticized. Hamilton (2018) proposes a regression filter as an alternative. Even if such regression filter overcomes the drawbacks of the H-P filter, it results to suffer some limitations, as discussed in Schüler (2018). Another procedure as in Bernard and Gerlach (1998) and Estrella and Hardouvelis (1991) is to set the GDP equal to unit during the quarters of recession indicated by the National Bureau of Economic Research (NBER).^1^ Applying the H-P filter to our data, we have encountered the limitations of this filter on the right tail. In Figure 1 each line represents a different size of the test set when splitting the entire time series into train and test sets. The red line shows the value of the cycle when the test set contains the 4 quarters of 2020, the green line does the job for 2 years (8 quarters) and so on. The feature that clearly emerges is that the values obtained through the filter are affected by the size of the test set. Using the test set with the last 4 and 8 quarters, the H-P filter assigns to the third quarter of 2020 a positive value. This means that the classification procedure on the filter generates those quarters as periods of growth (rather than recessions). As a result, the policymaker waste resources since the model is being fitted on unreliable data. When the test size is long enough, the filter provides the policymaker with appropriate values. Notice that the value obtained comparing the binary outcome derived from the H-P filter and the NBER data, that is the one for which the two time series match is 12 quarters in our example. We also control for the Subprime recession. Similarly, more than 4 quarters are required by H-P filter to match the NBER recession period for the second quarter of 2009, as shown in Figure 2. Since H-P filter cannot be considered reliable on the tails, the classification approach does not represent a trustworthy model for predicting growth (recession). Furthermore, another drawback of the classification is the loss of the quantitative information: the decision maker is provided with signal of growth or recession without any kind of information related to the magnitude of the event. We point out that neglecting such quantitative specification comes at a cost as the resulting classification will rely on biased trend-cycle decomposition and, therefore, be misleading. Instead, using predictions based on the actual value of the GDP, the benefit for the policymaker is to capture the intensity of the variation. In this way, the entity of the growth (recession) of the GDP assumes a real value that can be fundamental to address medium-term economic policies. In contrast to the cyclical indicator, this type of information gives the policymaker a wider set of possible actions than a binary pair (growth or recession), to better calibrate the reaction to expected changes in the GDP. For instance, the Federal Reserve System (FRS) may be interested in the GDP growth forecast with the aim to set the interest rate against any inflationary threats. On the one hand, when the forecast is based on classification, the only strategy the FRS can apply is to lower or raise the interest rate without knowledge of the value which is needed to set the policy. On the other hand, a quantitative information about the prediction of the GDP growth allows the FRS to optimally set the interest rate, following classical policies such as the Taylor rule (Taylor, 1993) or other rules, to respond to variation of the GDP. For all these reasons, we forecast the GDP with regression techniques.

### 2.2 GDP Forecasting Models

We explore different forecasting models to predict the United States GDP: KNN, AR, SARIMA, ARX, SARIMAX, and a particular specification of the classical linear regression model (LR). Let *t*_*i*_, *i* ∈ {1, 2, 3, 4} represents the *t*_*i*_ − *th* quarter of year *T* ∈ {1976, 1977, *…* , 2020}, so that *t* ∈ {1, *…* , 179} is the number of total quarters. Define $Y={\left\{y_{t}\right\}}_{t\in R^{+}}$ the time series of the *log* GDP. Let $Y^{d}={\left\{y_{t}-y_{t-d}\right\}}_{d,t\in R^{+}}$ be the *d* − *th* order difference between consecutive GDP time series observations. We denote with **X**_*n*_ = {**x**_*n*,*t*_} the time series of a generic set of *n* covariates with *n* ∈ {1, 2, 3, *…* , *N*}.

KNN. The KNN is a machine learning algorithm useful to solve both classification and regression problems (Wu et al., 2008) based on learning by analogy. We apply the KNN methodology to forecast univariate time series. The rationale behind the use of KNN for time series forecasting is that a time series may contain repetitive patterns. The *i*−th data point (target) can be described by a vector of *n* covariates $\left(x_{1}^{i},x_{2}^{i},\ldots ,x_{N}^{i}\right)$ that are the lagged values of the target $y_{i}^{1}$ . Consider a new observation, for example the next quarter $y_{t+1}^{1}$ to be predicted, whose covariates are known and denoted as $\left({\bar{x}}_{1},{\bar{x}}_{2},\ldots ,{\bar{x}}_{n}\right)$ . Note that there is a relationship between the covariates of the new observations that we want to forecast and the information that we have. The last targets are used as covariates of the new observation. Given that the minimum lag must be at least equal to the number of periods of forecast, in our analysis we use one covariate. For example, if the forecasting period is *h* = 10, the target $y_{t+1}^{1}$ will be described by the covariate *x*_*t*−10_. The KNN algorithm exploits the covariates of the new observation to find the *k* most similar training covariates according to a specified distance metric. In this study, we use as similarity metric the euclidean distance between the new observation *t* + 1 and the *i*−th training observation:

$$\sqrt{\overset{N}{\underset{n=1}{\sum }}{\left(x_{n}^{i}-{\bar{x}}_{n}\right)}^{2}}.$$ (1)

When predicting a new data point, the algorithm finds the *k* observed targets with covariates’ values (the *x* lagged quarters) closer to it. Then, it assigns to the new data point the average of the *k*’s target values. We use *tsfknn* library on the software *R* for the implementation (Martínez et al., 2019).

AR. The purely autoregressive process of order *p*, *AR*(*p*) satisfies the equation:

$$y_{t}=\overset{p}{\underset{i=1}{\sum }}\phi_{i}y_{t-i}+\varepsilon_{t}$$ (2)

where {*ɛ*_*t*_} is a white noise with *E* (*ɛ*_*t*_) = 0, $E\left(\varepsilon_{t}^{2}\right)=\sigma^{2}$ , *p* is the autoregressive order of the process with coefficients *ϕ*_*i*_. Thus, the *AR*(*p*) takes into account just the *p* previous periods, while the “new” part of *y*_*t*_, not linked to the past, is given by *ɛ*_*t*_.

ARX. The ARX model is an extension of AR that includes the time series of covariates **x**′_**k,t**_:

$$y_{t}=\overset{p}{\underset{i=1}{\sum }}\phi_{i}y_{t-i}+\overset{n}{\underset{k=1}{\sum }}\beta_{k}x_{k,t}+\varepsilon_{t}$$ (3)

SARIMA. The seasonal ARIMA (*p*, *d*, *q*) × (*P*,*D*,*Q*)_*S*_, or SARIMA, is a process that takes simultaneously into account two features of the observed time series: the correlation between consecutive values modelled by standard ARIMA and the correlation between observations that are far from each other that captures the seasonality. Formally, the ARIMA part of the model is defined as:

$$\begin{array}{c} y_{t}^{d}=\phi_{0}+\overset{p}{\underset{i=1}{\sum }}\phi_{i}y_{t-i}+b_{t}-\overset{q}{\underset{j=1}{\sum }}\theta_{j}b_{t-j}, \end{array}$$ (4)

where *p* is the autoregressive order of the process with coefficients *ϕ*_*i*_ and *q* is the order of the moving average process with coefficients *θ*_*i*_. Notice that in a standard ARIMA process *b*_*t*_ is white noise, whereas here it is not due to the existence of unexplained correlation that we model as follows:

$$\begin{array}{c} w_{t}=b_{t}-b_{t-D},\\ w_{t}=\overset{P}{\underset{i=1}{\sum }}\Phi_{i}w_{t-i\cdot S}+\varepsilon_{t}-\overset{Q}{\underset{j=1}{\sum }}\Theta_{j}\varepsilon_{t-j\cdot S}, \end{array}$$ (5)

where *D* represents the degree of the integration, *P* and *Q* are the seasonal orders of the autoregressive and moving average processes with coefficients Φ_*i*_ and Θ_*i*_, respectively, *S* is the seasonality, and $\varepsilon_{t}∼WN\left(0,\sigma_{\varepsilon }^{2}\right)$ . Using the lag operator *B* such that *By*_*t*_ = *y*_*t*−1_, then (4) and (5) define the SARIMA (*p*, *d*, *q*) × (*P*,*D*,*Q*)_*S*_ process written in compact form:

$$\phi \left(B\right)\Phi \left(B^{S}\right){\left(1-B\right)}^{d}{\left(1-B^{S}\right)}^{D}y_{t}=\phi_{0}+\theta \left(B\right)\Theta \left(B^{S}\right)\varepsilon_{t}.$$ (6)

SARIMAX. The SARIMAX model is an extension of SARIMA that includes the time series of covariates **x**′_**k,t**_:

$$\phi \left(B\right)\Phi \left(B^{S}\right){\left(1-B\right)}^{d}{\left(1-B^{S}\right)}^{D}y_{t}=\beta_{n}x_{n,t}^{\prime }+\theta \left(B\right)\Theta \left(B^{S}\right)\varepsilon_{t}.$$ (7)

Linear Regression. We specify the classical LR model as follows:

$$y_{i}^{1}=\beta_{0}+\beta_{1}x_{n,t}+\epsilon_{i},$$ (8)

where the dependent variable $y_{i}^{1}$ is the first order differentiated time series at time *t* and the covariates **x**_*n*,*t*_ are the variables at time *t* lagged of *h* periods where *h* defines the forecasting horizon. Despite the fact that LR does not account for the autoregressive component, which is typical in a time series, our specification is built in such a way that allows us to include a degree of temporal information.

### 2.3 Forecasting Strategies

We propose two different forecasting strategies with the aim of studying the accuracy of the GDP predictions when we include all the available information at present time. We also assess the magnitude of the precision for different forecasting horizons.

#### One-Step-Ahead Forecasting

The one-step-ahead forecasting strategy computes the forecast for one quarter ahead. This implies that the train set, that is the data used for the forecast, is reduced by one observation that corresponds to the forecasting horizon, which is our test set, and covariates have one period lag. We run the prediction of the GDP for each quarter of the period from the first quarter of 2019 to the last of 2020. In each forecast the test set moves back by one quarter and the train becomes one quarter shorter. It is important to highlight that the chosen out-of-sample forecasting horizon includes both 1 year of normal times (2019) and 1 year affected by the Sars-COVID-19 pandemic (2020). The forecasting methodology works as follow:

$$\begin{array}{cccccccc} \text{Train Set} & & & & & & & \text{Test Set}\\ y_{1,t_{2}},\ldots ,y_{T,t_{i}} & & & & & & & y_{T,t_{i+1}}\\ y_{1,t_{2}},\ldots ,y_{T,t_{i-1}} & & & & & & & y_{T,t_{i}}\\ \vdots & & & & & & & \vdots \\ y_{1,t_{2}},\ldots ,y_{T-h,t_{i}} & & & & & & & y_{T-h,t_{i+1}} \end{array}$$ (9)

#### Multi-Step-Ahead Forecasting

In the multi-step-ahead forecasting strategy predictions are run over the horizon that increases at each forecast. In this set up, the end point of the test period is set fixed to the last quarter of 2020 and the starting point moves back by one quarter each forecast. Both GDP and covariates enter the models with a lag equal to the forecasting horizon. The forecasting methodology works as follow:

$$\begin{array}{cccccccc} \text{Train Set} & & & & & & & \text{Test Set}\\ y_{1,t_{3}},\ldots ,y_{T,t_{3}} & & & & & & & y_{T,t_{4}}\\ y_{1,t_{4}},\ldots ,y_{T,t_{2}} & & & & & & & y_{T,t_{3}},y_{T,t_{4}}\\ \vdots & & & & & & & \vdots \\ y_{4,t_{2}},\ldots ,y_{T-3,t_{4}} & & & & & & & y_{T-2,t_{1}},\ldots ,y_{T,t_{4}} \end{array}$$ (10)

The maximum length of the forecasting horizon here considered is 12 quarters from the first quarter of 2018 to the last of 2020.

## 3 Empirical Analysis

### 3.1 Data

We measure the economic activity with the seasonally adjusted real U.S. GDP expressed in quarterly frequency and in log scale. The data span the period from second quarter of 1976 to fourth quarter of 2020, for an overall of 179 observations, and are available from the database of the Federal Reserve Bank of Saint Louis, Federal Reserve Economic Data, FRED.

Interest rates and proxies. Both short-term and long-term U.S. federal government interest rates are used in our study. Short-term interest rates are obtained from Treasury-Bills with maturities 3 and 6 months; long-term interest rates are from the U.S. government bonds with maturities of 2, 3, 5, 7 and 10 years. Drawing on Diebold et al. (2006) and Ang et al. (2006), we exploit an alternative representation of the yield curve through its latent factors, namely the level, slope, and curvature to capture the economic information contained in it. The level is computed taking the average of short-, medium- and long-term bonds; in our study we use the interest rates at 3 months, 2 and 10 years. The slope is the result of the difference between the shortest- and the longest-term yield, 3 months and 10 years. The curvature is estimated computing the double product of the medium-term yield minus the shortest- and the longest-term yield.

Macroeconomic variables. We extend the analysis introducing key observable macroeconomic variables. Following the existing literature (Ang et al., 2006; Diebold et al., 2006; Koop, 2013; Schorfheide and Song, 2015) we select the Consumer Price Index, Manufacturing Capacity Utilization, and Unemployment Rate to illustrate real economic activity whereas the Federal Funds rates proxies the monetary policy. The Manufacturing Capacity Utilization and the Consumer Price Index are differentiated to make the series stationary.

### 3.2 Models Fitting

*KNN*. Performing a grid search we find that optimal value of *k* is 2 for both forecasting strategies.

*AR*. We use stepwise procedure in order to choose the optimal autoregressive value of *p*, minimizing the *AIC* value.

*ARX*. The same methodology of AR has been applied to ARX.

*SARIMA*. With quarterly GDP data the seasonal period of the series is *s* = 4. Therefore, (11) becomes:

$$\Phi_{p}\left(B^{4}\right)\phi \left(B\right)\nabla_{4}^{D}\nabla^{d}y_{t}=\Theta_{Q}\left(B^{4}\right)\theta \left(B\right)w_{t}.$$ (11)

The orders *p*, *d*, *q* and *P*, *D*, *Q* are chosen performing stepwise search to minimize the AIC selection criterion.

*SARIMAX*. By (11), (7) becomes:

$$\Phi_{p}\left(B^{4}\right)\phi \left(B\right)\nabla_{4}^{D}\nabla^{d}y_{t}=\beta_{n}x_{n,t}^{\prime }+\Theta_{Q}\left(B^{4}\right)\theta \left(B\right)w_{t}.$$ (12)

Linear Regression. We fit a linear regression for each scenario and forecasting strategy. In the one-step-ahead forecasts the covariates have one period lag. In the multi-step-ahead the covariates have a lag equal to the length of the forecasting horizon, which increases at each forecast.

We include a set of covariates **x**′_**n,t**_ in LR, ARX and SARIMAX and study six different scenarios:

Scenario 1 = {Yield Curve};
Scenario 2 = {Yield Curve, Macro-variables};
Scenario 3 = {Macro-variables};
Scenario 4 = {Proxies};
Scenario 5 = {Macro-variables,Proxies};
Scenario 6 (Full) = {Yield Curve, Macro-variables, Proxies},

where the covariates for the yield curve are Treasury-Bills with maturities 3 and 6 months and 2, 3, 5, 7 and 10 years. Macro variables are Consumer Price Index, Manufacturing Capacity Utilization, Unemployment rate, and the Federal Funds rate. The proxies are the level, slope and curvature.

## 4 Results

### 4.1 Model Performances

The KNN model achieves the best forecasting results with respect to SARIMA and AR, specifications that do not include covariates, as reported in Table 1.

**TABLE 1:** Average MSE for all periods.

Strategy	SARIMA	KNN	AR
One-step	2,87e-03	1,73e-03	3,47e-03
Multi-step	3,84e-03	3,02e-03	4,18e-03

Other models that provide good forecasts are models that include covariates, namely SARIMAX, LR and ARX. We notice that both SARIMAX and LR tend to overestimate the GDP predictions. We also investigate the average of the predictions obtained with the two models (Mean LR-SARIMAX):

$$\hat{y}=\frac{1}{2}\left({\hat{y}}_{t,LR}+{\hat{y}}_{t,SARIMAX}\right).$$ (13)

Table 2 reports the average MSE. Among all the models, KNN provides the best forecasts. SARIMAX and ARX are able to better predict the GDP one-step-ahead when interest rates (Scenario 1) and proxies (Scenario 4) are considered as covariates. This finding remains true also when forecasting with the multi-step-ahead strategy.

**TABLE 2:** Comparison of average MSE in the two strategies considered: one-step- vs. multi-step-ahead.

	SARIMAX	LR	Mean LR-SARIMAX	ARX
One step				
Scenario 1	2,44e-03	1,93e-03	2,15e-03	3,57e-03
Scenario 2	6,19e-03	2,41e-03	3,96e-03	7,81e-03
Scenario 3	5,90e-03	2,38e-03	3,84e-03	5,92e-03
Scenario 4	2,86e-03	1,89e-03	2,31e-03	3,45e-03
Scenario 5	6,10e-03	2,43e-03	3,96e-03	6,66e-03
Scenario 6	6,14e-03	2,41e-03	3,94e-03	7,99e-03
Multi step				
Scenario 1	3,66e-03	2,50e-03	3,01e-03	4,29e-03
Scenario 2	3,99e-03	2,48e-03	3,13e-03	5,24e-03
Scenario 3	4,05e-03	2,38e-03	3,08e-03	4,69e-03
Scenario 4	3,97e-03	2,40e-03	3,06e-03	5,24e-03
Scenario 5	4,57e-03	2,37e-03	3,26e-03	4,84e-03
Scenario 6	3,97e-03	2,48e-03	3,13e-03	4,86e-03

Overall, the one-step-ahead predictions with Scenarios 1 and 4 are the most accurate, whereas the multi-step-ahead forecast with macro variables (Scenario 3) contributes to improve the predictions the most. The Mean LR-SARIMAX performs equally likely as the SARIMAX.

### 4.2 Out-of-Sample One-Step-Ahead Forecasting Performance

**FIGURE 3:**
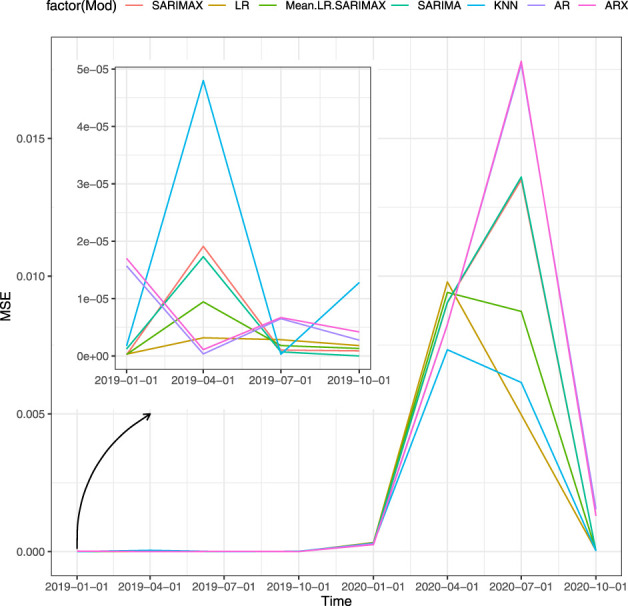
MSE of the models for one-step-ahead, Scenario 4, sensitiveness to the pandemic shock.

Table 3 displays the prediction accuracy for the forecasting horizon of Scenario 4 (proxies). Figure 3 shows the accuracy, in terms of MSE, that fitted models achieve in each forecast horizon in the one-step-ahead strategy. The clear pattern that emerges is the change in the best performing model due to the COVID-19 shock. Specifically, models with the autoregressive component perform better before the second quarter of 2020 while the other models result to better respond to COVID-19. On the one hand, the KNN provides the best out-of-sample prediction for the second quarter of 2020 that corresponds to the beginning of the pandemic outbreak. On the other hand, SARIMAX is more accurate in normal periods as it achieves the lowest forecast error for the first quarter of 2019. The same holds for both AR and ARX which are the most accurate in the second quarter of 2019. SARIMA is the best performing model for the fourth quarter of 2019.

**TABLE 3:** MSE One-step-ahead forecast, Scenario 4.

Dates	SARIMAX	LR	Mean LR-SARIMAX	SARIMA	KNN	AR	ARX
2020-10-01	2,27e-05	3,51e-05	2,86e-05	1,85e-05	2,56e-05	1,54e-03	1,30e-03
2020-07-01	1,35e-02	4,97e-03	8,72e-03	1,36e-02	6,14e-03	1,77e-02	1,78e-02
2020-04-01	9,03e-03	9,79e-03	9,41e-03	9,05e-03	7,33e-03	8,25e-03	8,24e-03
2020-01-01	2,85e-04	3,31e-04	3,07e-04	3,07e-04	2,97e-04	2,83e-04	2,54e-04
2019-10-01	8,94e-07	1,81e-06	1,31e-06	2,02e-09	1,28e-05	2,76e-06	4,19e-06
2019-07-01	1,03e-06	2,84e-06	1,82e-06	7,14e-07	3,07e-07	6,50e-06	6,70e-06
2019-04-01	1,91e-05	3,17e-06	9,44e-06	1,73e-05	4,80e-05	3,58e-07	1,11e-06
2019-01-01	2,10e-07	3,27e-07	2,65e-07	1,23e-06	1,75e-06	1,57e-05	1,70e-05

The second best forecasting model is the LR. As shown in Figure 3, it performs well on the whole forecasting horizon. Looking at single scenarios that include the LR outperforms the other models, confirming the forecasting-power of the yield curve in predicting the GDP.

### 4.3 Out-of-Sample Multi-Step-Ahead Forecasting Performance

**FIGURE 4:**
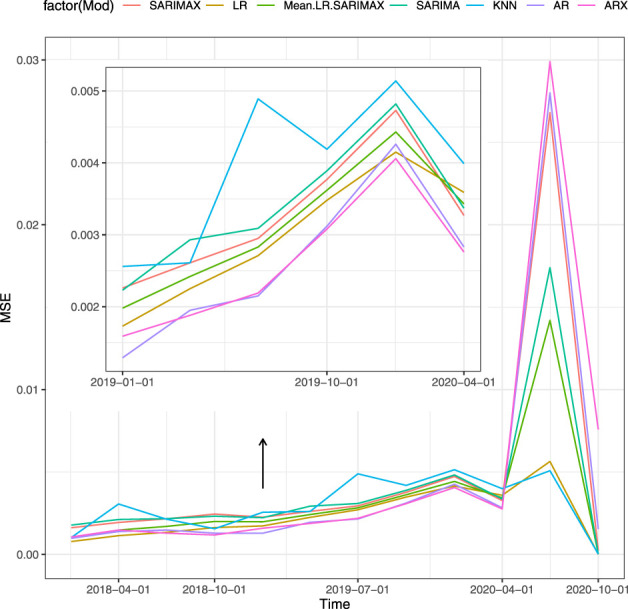
MSE of the models for multi-step-ahead, Scenario 5, sensitiveness to the pandemic shock.

Table 4 shows the results of the second type of forecasting strategy for the Scenario 5 (proxies and macro variables). Figure 4 shows the MSE of the models for each forecasting horizon. The change occurs also for the multi-step-ahead strategy and the time series models loss the most in terms of performance after the second quarter of 2020. The best overall performance is achieved by the LR with this specification. We highlight that such set of covariates performs better than other combinations, namely Scenario 1, 2, 3, 4, and 6. The average MSE with Scenario 5 is the lowest among models with and without covariates. This result holds true for both periods of stability and crisis. A possible justification lies in the fact that the LR does not include the autoregressive term of the GDP that may affect the prediction performance. Indeed, the macro variables may be more reactive improving the prediction compared to autoregressive models.

**TABLE 4:** MSE multi-step-ahead forecast, Scenario 5.

Dates	SARIMAX	LR	Mean LR-SARIMAX	SARIMA	KNN	AR	ARX
2020q4	2,42e-04	5,00e-06	4,40e-05	1,80e-05	2,56e-05	1,54e-03	7,58e-03
2020q3–2020q4	2,68e-02	5,64e-03	1,42e-02	1,74e-02	5,08e-03	2,80e-02	2,99e-02
2020q2–2020q4	3,27e-03	3,59e-03	3,43e-03	3,37e-03	3,99e-03	2,83e-03	2,76e-03
2020q1–2020q4	4,73e-03	4,15e-03	4,43e-03	4,82e-03	5,14e-03	4,26e-03	4,06e-03
2019q4–2020q4	3,77e-03	3,48e-03	3,62e-03	3,89e-03	4,19e-03	3,12e-03	3,08e-03
2019q3–2020q4	2,95e-03	2,71e-03	2,83e-03	3,09e-03	4,89e-03	2,15e-03	2,19e-03
2019q2–2020q4	2,61e-03	2,25e-03	2,42e-03	2,93e-03	2,61e-03	1,95e-03	1,88e-03
2019q1–2020q4	2,26e-03	1,73e-03	1,98e-03	2,23e-03	2,56e-03	1,29e-03	1,59e-03
2018q4–2020q4	2,45e-03	1,63e-03	2,00e-03	2,32e-03	1,56e-03	1,29e-03	1,18e-03
2018q3–2020q4	2,16e-03	1,34e-03	1,70e-03	2,17e-03	2,14e-03	1,48e-03	1,29e-03
2018q2–2020q4	1,94e-03	1,14e-03	1,48e-03	2,12e-03	3,06e-03	1,39e-03	1,48e-03
2018q1–2020q4	1,62e-03	7,83e-04	1,04e-03	1,78e-03	1,03e-03	9,78e-04	1,06e-03

## 5 Conclusion

In this article, we provide a comparison of the predictive ability of time series, linear regression, and machine learning models to forecast the U.S. GDP. We discuss the benefit for the policymaker of a regression approach compared to the classification to address medium-term policies. Moreover, we evaluate two different strategies of forecasting, one-step-ahead and multi-step-ahead, considering the self-explanatory power of GDP and the importance of financial and macro-economic variables as predictors. On the one hand, the machine learning KNN achieves the best performance for the one-step-ahead strategy, providing evidence that in the subsequent horizon the exploitation of repetitive patterns in the GDP increases the forecast. On the other hand, it loses predictive power when the forecast is performed for a longer horizon. SARIMA performs poorly in the one-step-ahead and multi-step-ahead strategies. Including covariates, SARIMAX obtains a lower error in the one-step-ahead strategy especially with the Treasury-Bills with maturities 3 and 6 months and 2, 3, 5, 7 and 10 years (Scenario 1). ARX achieves the best forecasting performance in one-step-ahead with proxies (Scenario 4) and yield curve (Scenario 1). Considering the multi-step-ahead accuracy, the yield curve has proved to be the best predictor to be paired with this model. Surprisingly, the LR achieves the best performance in the multi-step-ahead forecast using proxies for the yield curve and macro variables (Scenario 5). Moreover, it achieves the second-best performance in the one-step-ahead strategy using only the proxies as predictors and confirming the strong predictive power of the yield curve for the GDP. In general, we find that a switch occurs in terms of forecasting performances, both for one and multi-step-ahead (see Figures 3, 4), between models which have the autoregressive component and models without it. Before the cutoff, the pandemic outbreak in our study, time series models perform better but after that event LR and KNN outperform the other approaches. The results of our analysis suggest the use of the KNN model for one-step-ahead forecasts and that of LR with the use of financial variables for multi-step-ahead forecasts. We propose to overcome the trade-off between accuracy in the estimates and the forecasting horizon, considering the two forecasting strategies which are not mutually exclusive. Indeed, the benefit of a continuous forecasting of both one-step-ahead and multi-step-ahead allows the decision-maker to have two useful instruments: on the one hand the multi-step provides a long-term vision for planning in advance investments, monetary policy, etc., on the other hand the one-step-ahead might tip the scale for possible refinement around the decision taken. There are many possible avenues for future works. A desirable address is to develop a model that includes the international bond yield curve (Byrne et al., 2019), macro variables, and the GDP of countries the United States trade with.

## Data Availability

The data analyzed in this study are freely available at the Federal Reserve Bank of Saint Louis, Federal Reserve Economic Data (FRED) website, https://fred.stlouisfed.org/. The interested reader may use the provided link to FRED to explore the data. Any further inquiries can be directed to the corresponding author.

## Footnotes

1 NBER considers as recessions two consecutive quarters of negative GDP growth
